# Functional Topography of Human Corpus Callosum: An fMRI Mapping Study

**DOI:** 10.1155/2013/251308

**Published:** 2013-02-14

**Authors:** Mara Fabri, Gabriele Polonara

**Affiliations:** ^1^Sezione di Neuroscienze e Biologia Cellulare, Dipartimento di Medicina Sperimentale e Clinica, Università Politecnica delle Marche, 60020 Ancona, Italy; ^2^Sezione di Scienze Radiologiche, Dipartimento di Scienze Cliniche Specialistiche e Odontostomatologiche, Università Politecnica delle Marche, 60020 Ancona, Italy

## Abstract

The concept of a topographical map of the corpus callosum (CC) has emerged from human lesion studies and from electrophysiological and anatomical tracing investigations in other mammals. Over the last few years a rising number of researchers have been reporting functional magnetic resonance imaging (fMRI) activation in white matter, particularly the CC. In this study the scope for describing CC topography with fMRI was explored by evoking activation through simple sensory stimulation and motor tasks. We reviewed our published and unpublished fMRI and diffusion tensor imaging data on the cortical representation of tactile, gustatory, auditory, and visual sensitivity and of motor activation, obtained in 36 normal volunteers and in 6 patients with partial callosotomy. Activation foci were consistently detected in discrete CC regions: anterior (taste stimuli), central (motor tasks), central and posterior (tactile stimuli), and splenium (auditory and visual stimuli). Reconstruction of callosal fibers connecting activated primary gustatory, motor, somatosensory, auditory, and visual cortices by diffusion tensor tracking showed bundles crossing, respectively, through the genu, anterior and posterior body, and splenium, at sites harboring fMRI foci. These data confirm that the CC commissure has a topographical organization and demonstrate that its functional topography can be explored with fMRI.

## 1. Introduction

The corpus callosum (CC) connects the cerebral hemispheres and provides for interhemispheric integration and transfer of information. Ever since electrophysiological recording from callosal fibers showed somatosensory receptive fields in the anterior portion of the cat commissure [1, 2] and visual inputs to the splenium [3, 4], it was hypothesized that the CC was endowed with a topographical organization. Subsequent electrophysiological [5] and neuroanatomical findings [6, 7] obtained from nonhuman primates after selective cortical ablation or tracing injections, plus a vast body of data ranging from postmortem investigations [8] to studies of patients with CC lesions or callosal resection (split-brain subjects; [9]; see [10–12] for a review), lent further support to the notion. Such organization seems to result in modality-specific regions [13], where the anterior callosal fibers interconnecting the frontal lobes transfer motor information and posterior fibers, which connect the parietal, temporal, and occipital lobes bilaterally, are responsible for the integration of somatosensory (posterior midbody), auditory (isthmus), and visual (splenium) information.

The hypothesis was finally confirmed by studies largely carried out in subjects with callosal resection. Functional magnetic resonance imaging (fMRI) investigations of split-brain patients by our group [12, 14, 15] provided evidence that interhemispheric transfer in the tactile modality is likely mediated by fibers running through the posterior part of the callosal body, thus confirming that the posterior midbody is the tactual channel. A recent study of nonepileptic patients with transection of different portions of the anterior CC performed to remove cysts [9] provided further confirmation demonstrating that the middle part of the genu is involved in motor coordination and the anterior portion of the body in the transfer of simple somesthetic information.

Investigations of other sensory modalities have shown that the splenium is crucial for the interhemispheric transfer of visual [16] (see data and literature in [17]) as well as auditory information [18, 19].

The advent of functional MRI subsequently made it possible to study the intact brain noninvasively. In recent years an increasing number of fMRI studies have described white matter (WM) activation in the anterior CC during behavioral tasks involving interhemispheric transfer [20–23] as well as during voluntary swallowing, which is not a specific interhemispheric transfer task [24]. A BOLD (blood oxygenation level dependent) effect was also detected in the posterior callosal region (isthmus and splenium) during an interhemispheric transfer task based on the crossed nature of the visual and motor systems which assumed that information must cross the CC to elicit a behavioral response (“crossed condition”; [23]). The anterior CC has been seen to be involved in transferring information between prefrontal and premotor regions, and the posterior CC in information transfer between parietal, occipital, and temporal cortices [25–28].

These reports, documenting callosal functional activation during behavioral tasks, prompted a review of our published and unpublished fMRI data—obtained from studies of the cortical representation of gustatory, tactile, auditory, and visual sensitivity and of motor activation in normal subjects—to establish whether the BOLD effect could be detected in callosal WM and whether the activation foci evoked by a range of simple sensory stimuli and motor tasks were consistent with a topographical organization. Indeed activation foci were consistently present in discrete CC regions: anterior (taste stimuli), central (motor tasks), central and posterior (tactile stimuli), isthmus (auditory stimuli) and splenium (visual stimuli), demonstrating that the functional topography of the CC can be explored with fMRI [29]. Very recently we administered the peripheral sensory stimulation protocols previously applied to study normal subjects (described in [29]) to patients with partial callosotomy (Polonara et al., submitted). The results were analyzed for evidence of a specific BOLD effect in the extant callosal portions, further to document the notion of a functional CC map. Diffusion tensor imaging (DTI) data were acquired in control subjects and in patients and analyzed to establish whether CC activation was colocalized with tracts seeded from activated clusters in cortical areas involved by specific sensory stimuli.

In this paper, the results obtained in normal subjects and in callosotomy patients are presented together to provide an overview of the functional organization of the CC.

## 2. Methods

### 2.1. Subjects

The data were collected from 36 normal volunteers (age range 22–51 years, 20 women; Table 1) and 6 callosotomy patients (age age range 22–51 years, 2 women; Table 2) during studies of gustatory, tactile, auditory, visual, and motor cortical representation. The callosotomy, performed to treat drug-resistant epilepsy, involved the anterior CC in 4 subjects (L.M., O.T., R.V., and D.B.), the posterior CC in one (M.C.) and the central CC in the last patient (P.F.; Table 2). All subjects (controls and patients) gave their informed consent to participate in the study, whose experimental protocol was approved by the local ethics committee. Handedness was evaluated by the Oldfield inventory [30]. Tactile stimulation was applied to 22 normal subjects and to all 6 patients, gustatory stimulation to 13 controls and 4 patients, and visual stimulation to 14 control subjects and 5 patients; one control subject and 3 patients also received auditory stimulation. Seven normal subjects were scanned while performing the motor tasks (Table 1). All stimuli were presented according to a block-design protocol that alternated periods of rest and of stimulation. Taste and touch stimuli were applied to the left or right side in different sessions; visual stimuli were presented to the left or right visual field (LVF; RVF) in the same session, or to the central visual field (CVF) in a separate session. Auditory stimuli were presented alternatively to the left and right ear in the same session (2 patients) or bilaterally to both ears (the other patient and the control subject).

### 2.2. Imaging Protocols

For all investigations, subjects were placed in a 1.5 Tesla (T) scanner (Signa Excite NV/i CV/i, General Electric Medical System, Milwaukee, WI, USA) equipped with 50 mT/m gradients, with their head restrained within a circularly polarized head coil. They were instructed to keep their eyes closed and find a comfortable position and relax, avoiding even minimal movement; their ears were plugged.

#### 2.2.1. Functional Imaging

An identical 4-step experimental procedure was applied in all cases. In the first step, an anatomical three-plane localizer (2D SPGR, TR 120 ms, TE 15 ms, Flip Angle 70°, FOV 23 × 23 cm, slice thickness 5 mm, Matrix 256 × 256, 1 Nex, scan time 31 s) was acquired. The second step entailed acquisition of a 3D data set (IR Prep Fast SPGR; TR 15.2 ms, TE 6.9 ms, TI 500 ms, Flip Angle 15°, FOV 29 × 29 cm, slice thickness 1 mm, Matrix 288 × 288, 1 Nex, and scan time 8 : 20 min). The third involved acquisition of 10 (20 in more recent studies) contiguous 5-mm-thick axial or oblique functional images with a single-shot T2*-weighted gradient-echo EPI sequence (TR 3000 ms, TE 60 ms, Flip Angle 90°, FOV 28 × 21 cm, Matrix 96 × 64, 1 Nex, scan time 5 : 12 min). In the fourth step high-resolution axial (or oblique) anatomical images were acquired from 10 selected planes (2D SPGR, TR 100 ms, TE 12 ms, Flip Angle 70°, FOV 28 × 21 cm, thickness 5 mm, Matrix 256 × 256, 1 Nex, scan time 3 : 17 min for 10 images) so that functional activation images could be superimposed onto the anatomical landmarks, to show blood vessels which are possible sources of BOLD signal. In more recent studies the images were acquired from 20 rather than 10 axial planes.

One thousand (or 2000) axial or oblique functional images (100 per section, 1 image/3 s) were acquired during the stimulation cycle from the 10 contiguous 5-mm-thick axial sections obtained from the 10 (or 20) previously selected planes. Consecutive images from each section were examined in cine mode for head movements (see [31] for a review). Stimulation cycles in which head motion was detected were discarded. Functional images were obtained with the BOLD method. The axial planes were orthogonal to both the sagittal and the coronal planes and allowed the identification of the central sulcus, postcentral gyrus, sylvian sulcus, frontal and parietal operculum, calcarine fissure, and primary visual cortex (VI).

#### 2.2.2. Diffusion Tensor Imaging

When DTI became available at our institution a fifth step was added. Data were acquired from 17 control subjects and all 6 patients to study the connections between activated cortical areas and establish whether CC regions crossed by interhemispheric fibers reconstructed by diffusion tensor tracking (DTT) were linked to areas where the BOLD effect had been observed. For the DTI study a series of oblique axial images was obtained using a single shot spin-echo echo-planar sequence with a diffusion-sensitizing gradient. Diffusion was measured along 25 noncollinear directions. The *b* value was 1000 s/mm^2^. Acquisition parameters were TR 6500 ms, TE 76.2 ms, Matrix 128 × 128, FOV 26 × 26 cm, slice thickness 5.0 mm, interslice gap 1.0 mm, Nex 2, and scan time 5 : 51 min.

### 2.3. Stimulation Protocols

For *taste stimulation* 13 normal volunteers received gustatory stimuli. Tastants were 1 M NaCl for the salty stimulus (10 subjects), 10% sucrose for the sweet stimulus (6 subjects), and 0.002 M quinine chloride for the bitter stimulus (3 subjects). A neutral stimulus (distilled water) was also applied in 7 subjects, twice or 4 times as appropriate. There were two taste stimulation protocols, each lasting 5 min: each sapid stimulus was presented twice under protocol 1 (60 s rest, 30 s stimulation, 90 s rest, 30-s stimulation, 90 s rest) and 4 times under protocol 2 (30 s rest, 15 s stimulation, 45 s rest, 15 s stimulation, 45 s rest). In the 4 patients receiving gustatory stimuli 1 M NaCl solution was applied to each side of the tongue in different scans, according to protocol 2. Stimulation was not applied during rest periods.

Seven subjects were scanned while performing *motor tasks* (two block-design protocols envisaging 10 alternate 30-s periods of rest and stimulation; Table 1) that consisted of alternate flexion and distension of the fingers of one hand (motor protocol 1) or in simultaneous haptic manipulation of an object held in both hands (motor protocol 2).

*Tactile stimulation* of one or more body areas involved 22 normal volunteers. Stimuli were applied to the right or left body side, or to both sides (Table 1) by rubbing the skin with a soft cotton pad (trunk), a soft sponge (face), or a rough sponge (hand, foot, and limbs) at a frequency of 1 Hz (Table 1). Three stimulation paradigms were used, each lasting 5 min. Touch protocol 1 was used to stimulate a single body region and was consisted of ten 30-s alternating periods of rest and stimulation. Touch protocol 2 envisaged stimulation of two body regions in the same scanning session and included 20 alternating periods of rest and stimulation, each lasting 15 s; during stimulation periods, one of the two body regions was stimulated alternately (e.g., hand and foot), enabling a larger number of regions to be stimulated. Some of these subjects (Al.M., G.M., A.V., E.B., M.S., and Lu.A.) also participated in a study of taste sensitivity and received tactile stimulation of the tongue for comparison with taste stimulation. In this case, tactile stimuli were applied with the same timing of taste protocol 2. As regards patients, *tactile stimuli* were applied to the left and/or right hand (6 patients; Table 2) by rubbing the palm with a rough sponge at a frequency of 1 Hz (Table 1) using a 5 min protocol consisting of ten 30-s alternating periods of rest and stimulation.

*Auditory stimulation* involved 1 control and 3 patients (Tables 1 and 2). For 2 patients the stimuli were pieces of classical music played alternately to the left (L) or right (R) ear, according to a block-design experimental paradigm consisting of 20 alternating periods of rest (15 s) and stimulation (15 s). For the third patient and the control subject auditory stimuli were Italian words spoken to both ears at the same time, according to a block-design experimental paradigm consisting of 10 alternating periods of rest (30 s) and stimulation (30 s). Stimuli were administered by means of fMRI-compatible headphones (Resonance Technology Inc., Northridge, CA, USA).

*Visual stimulation* involved 14 volunteers and 5 patients (Table 1). Stimuli were generated using an in-house-developed software and projected into an fMRI-compatible goggle system (Resonance Technology Inc., Northridge, CA, USA). The block-design experimental paradigm consisted of 20 alternating periods of rest (15 s) and stimulation (15 s). A black and white checkerboard (amplitude: 6°; virtual Cartesian distance from viewer's eyes: 75 cm) was flashed (1 Hz) to the CVF (9 subjects) or to the lateral VF (5 subjects), alternately to the left (L) or right (R) periphery (polar distance from the center of the display: 12°). In 2 subjects the stimulus was flashed simultaneously to the LVF and RVF. The same types of stimuli were also presented to the 5 callosotomy patients: to the CVF in 3 and to the lateral VF in 5 patients (Table 2), alternately to the LVF and RVF periphery. All subjects were asked to fixate a cross in the centre of the display during functional image acquisition; eye movements were monitored with an internal camera.

### 2.4. Data Analysis

After each experimental session images were transferred to a Unix workstation (General Electric Advantage Windows 4.2) and then to a personal computer. Data were analyzed with the BrainVoyager QX (BV QX) software (Brain Innovation, Maastricht, The Netherlands).

#### 2.4.1. Functional Imaging

The first two images of each functional series were discarded to take into account signal intensity variations due to progressive saturation. Functional and anatomical data were converted to BV internal data format and preprocessed [32]. Slice scan time correction and head motion correction were applied to the functional data of each subject. 3D anatomical data were preprocessed with intensity inhomogeneity correction and spatial transformation, and then transformed to Talairach standard space [33]. Coregistration of functional and anatomical data resulted in a normalized 4D volume time course data, which allowed the transformation of functional time series into Talairach space and identification of the position of activated areas using the Talairach coordinate system.

Statistical analysis was performed for each subject using the general linear model (GLM). This model aims to predict the variation of a dependent variable (the fMRI time course) in terms of linear combination. The predictor time course was convolved with a standard hemodynamic response function (HRF) to account for the hemodynamic delay.

Each subject's entire CC was examined during each task. Activation foci observed in callosal WM were studied by selecting regions of interest (ROIs) in the CC portions harboring activation foci. When the signal was significantly greater than the baseline (*P* < 0.05) and it correlated temporally with the stimulation pattern, activation was assumed to be evoked by the peripheral stimulus and the focus was included in the counts. Only foci whose voxels were all superimposed on the CC were counted as “callosal.” Each subject performed a given task only once. However, if foci were detected both in the anterior and posterior CC, data were assigned to different data groups.

Each stimulation protocol was administered only once. Foci whose *y* coordinate was greater (more positive) than −10 were considered “anterior” and those whose *y* coordinate was equal to or less than −10 were considered posterior. The value of −10 was equally distant from the most anterior and the most posterior foci. Averaged time courses were calculated within each ROI to show the mean BOLD signal change due to the stimulus. The BOLD signal change was expressed as a percentage of baseline.

The Wilcoxon test was applied to the *y* coordinates of the callosal WM foci evoked by the different tasks.

#### 2.4.2. Diffusion Tensor Imaging

For DTI data analysis, images were transferred to the Unix workstation for postprocessing with Functool 3.1.22 (General Electric Medical Systems, Milwaukee, WI, USA). EPI distortion was corrected automatically. Diffusion eigenvectors and eigenvalues calculated from the diffusion tensor represented the main direction of diffusion and the associated diffusivity. Anisotropy was calculated by using orientation-independent fractional anisotropy (FA). The FiberTrak option allows Functool to create 2D color orientation maps, 2D color eigenvector maps, and 3D tractography maps. The 3D volume viewer enables areas of high FA to be displayed as 3D images. The anisotropy threshold for termination of tracking was 0.18.

For tractography, CC fiber tracts were reconstructed starting from voxels with an FA > 0.18 in the different axial planes, according to the main vector, up to those with an FA < 0.18, or up to a maximum step size of 160 *μ*m (the length threshold of the fibers generated by the tracking).

ROIs were selected in brain regions including activated cortical areas to track the nerve fibers arising from individual ROIs. ROIs selected in different cortical regions had different sizes (gustatory cortex, 330 mm^2^; somatosensory cortex, 160 mm^2^; visual cortex, 105 mm^2^) due to the different size of the activation foci. As a control, ROIs measuring 26–50 mm^2^ were placed in CC portions displaying a BOLD effect, to establish which cortical areas were connected by the fibers coursing through them and whether they were the same areas activated by peripheral sensory stimulation. All ROIs were defined manually on color-coded maps of the main diffusion directions.

## 3. Results

### 3.1. Functional Activation of the Corpus Callosum 

*Taste stimulation (13 normal subjects and 4 callosotomy patients)*

**Figure 1:**
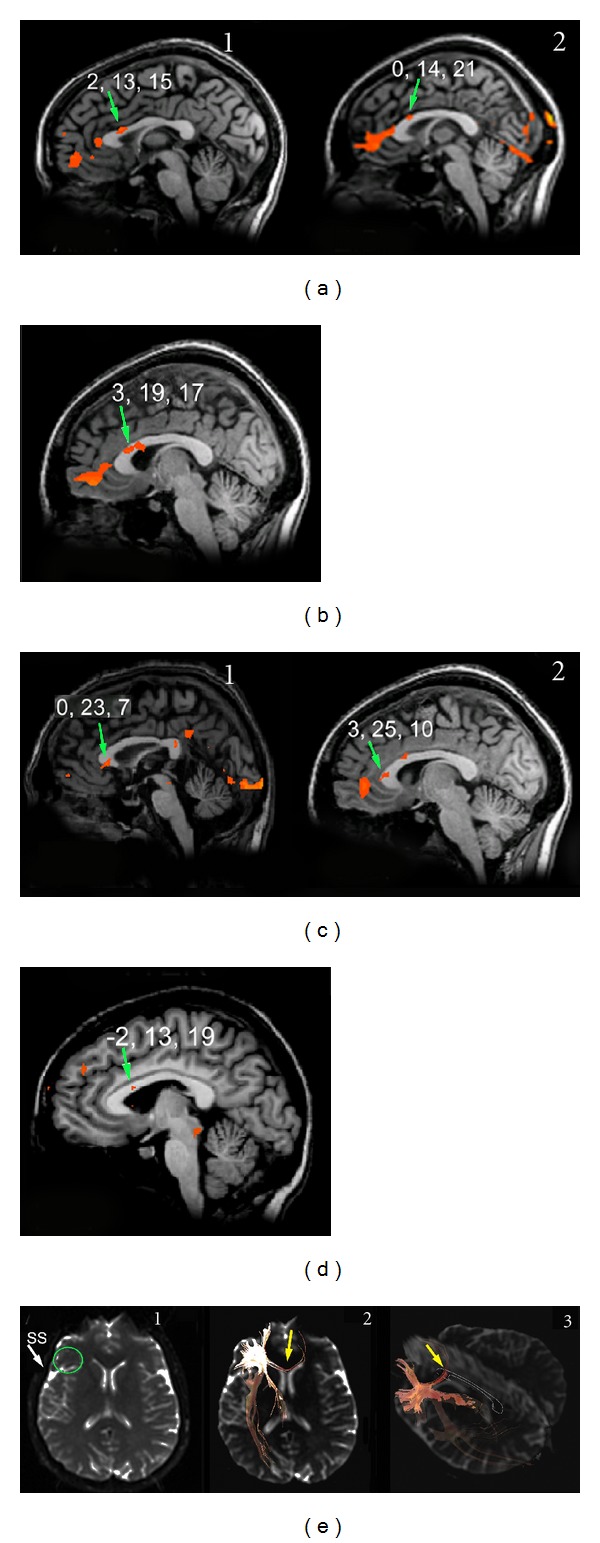
Callosal BOLD effect evoked by different tastants in normal subjects: (a) callosal foci evoked by the salty stimulus in 2 subjects (Al.M. and E.B.), both displaying activation in the anterior portion of the CC (green arrows), (b) callosal foci evoked by the neutral stimulus in subject E.B. displaying the activation in the anterior portion of the CC (green arrow), (c) callosal focus evoked by the sweet stimulus in 2 subjects (L.A. and E.B.), both exhibiting activation in the foremost portion of the corpus callosum (green arrows); a posterior focus, likely in the splenium, is also visible in subject L.A. (1), (d) callosal foci evoked by the bitter stimulus in subject L.E., displaying activation in the anterior portion of the CC (green arrow), and (e) callosal fibers arising from a cortical ROI selected in area GI (1, green circle) cross through the anterior CC (2 and 3, yellow arrows). Numbers on each brain represent the Talairach coordinates (*x*, *y*, *z*) of each focus (green arrows). On DTI images, a thin white line demarcates the CC. SS, Sylvian sulcus.

Unilateral taste stimulation applied to each side of the tongue in separate scanning sessions (Table 1) induced bilateral activation of the primary gustatory area (GI) in the frontoparietal operculum in all 13 subjects [34]. The same stimuli also evoked one or two callosal foci that were found most frequently in the anterior CC (Figures 1(a)–1(d)); foci were sometimes detected also in the posterior CC (Figure 1(c)1). Both anterior and posterior foci were seen after sweet stimulation in one subject and after bitter stimulation in two (Table 3). The anteroposterior Talairach coordinate (*y*) of the posterior foci ranged from −31 to −40, and that of the anterior foci from 9 to 23. The mean *y* values of the anterior foci activated by the sweet stimulus appeared to be significantly more anterior than those evoked by salty and bitter tastants (Table 3). In these subjects interhemispheric fibers crossing through the anterior CC were visible (Figure 1(e)), as detailed below.

**Figure 2:**
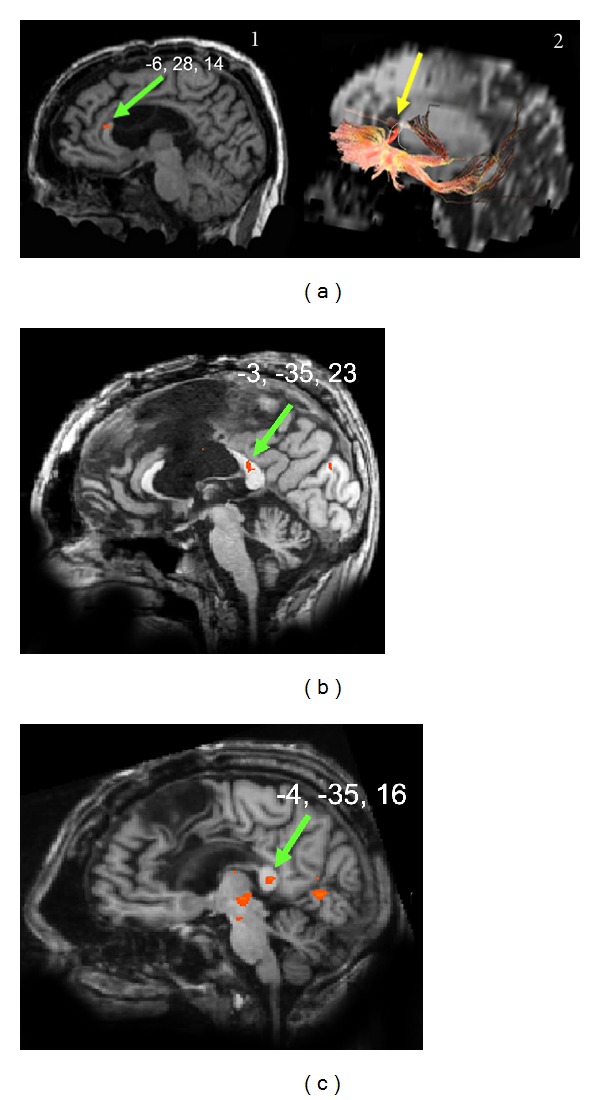
Activation evoked by taste stimulation in patient M.C. Bilateral cortical activation in the frontoparietal operculum (not shown) was seen in this subject, who also displayed a callosal focus ((a)1, green arrow). Fibers arising from the activated cortex cross through the anterior CC ((a)2, yellow arrow), (b) and (c) activation foci evoked in the splenium by taste stimuli in patients P.F. and L.M., respectively. No callosal fibers connecting taste areas crossed this splenium. On DTI images, a thin white line outlines the spared CC ((a)2). Numbers on each brain represent the Talairach coordinates (*x*, *y*, *z*) of each focus (green arrows).

Also in the 4 patients a salty stimulus applied to each side of the tongue in separate scanning sessions (Table 2) induced bilateral activation of area GI [34]. In the 2 subjects whose anterior CC was spared (M.C. and P.F.) the same stimulus also evoked a callosal focus in the anterior CC (Figure 2(a)1). The mean *y* coordinate of the callosal foci was 22.5 (Table 3; *P* < 0.05). In these 2 patients interhemispheric fibers crossing through the spared anterior CC were visible (Figure 2(a)2), as detailed below. In the other 2 patients and in P.F. an activation focus was also noted in the posterior CC (mean *y* coordinate: −35.33; Table 3; Figures 2(b) and 2(c)). The coordinates of both anterior and posterior foci evoked by taste stimulation were similar to those observed in control subjects [29].

*Motor Activation (7 Normal Subjects)*

**Figure 3:**
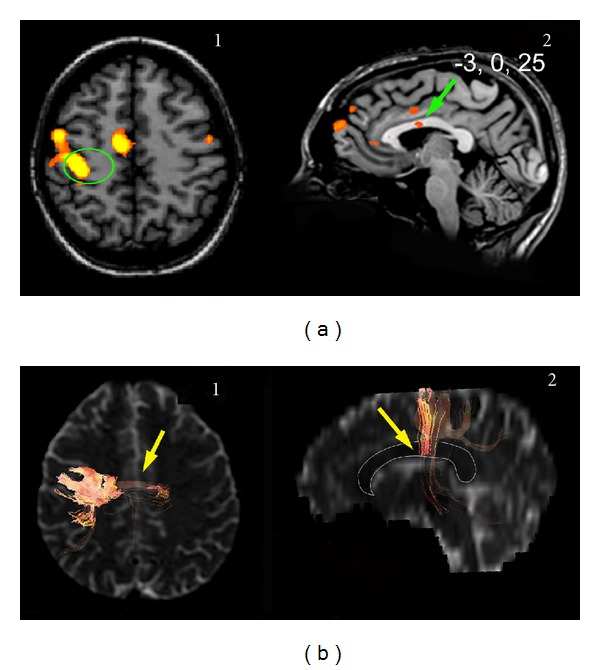
Callosal BOLD effect evoked by hand motor activation in a normal subject (G.M.). Activation was observed in the primary motor cortex ((a)1) and in the central part of the CC ((a)2), (b) callosal fibers arising from a cortical ROI selected in area MI ((a)1, green circle) cross through the central CC ((b)1 and (b)2, yellow arrows). On DTI images, a thin white line demarcates the CC. Numbers on the brain represent the Talairach coordinates (*x*, *y*, *z*) of the focus (green arrows).

Alternate flexion and distension of the fingers of a hand and object manipulation with both hands evoked foci in cortical motor areas and in the middle portion of the CC (Figure 3(a)2)—where anterior foci evoked by tactile hand and foot stimulation were also observed—in all 7 subjects (Table 3). The values of the y coordinate (1 to −23) overlapped with those of the anterior somatosensory foci. The interhemispheric fibers linking the activated motor cortical areas crossed through the middle part of the CC (Figures 3(b)1 and 3(b)2) at sites harboring the BOLD foci.

*Tactile Stimulation (22 Normal Subjects and 6 Callosotomy Patients)*

**Figure 4:**
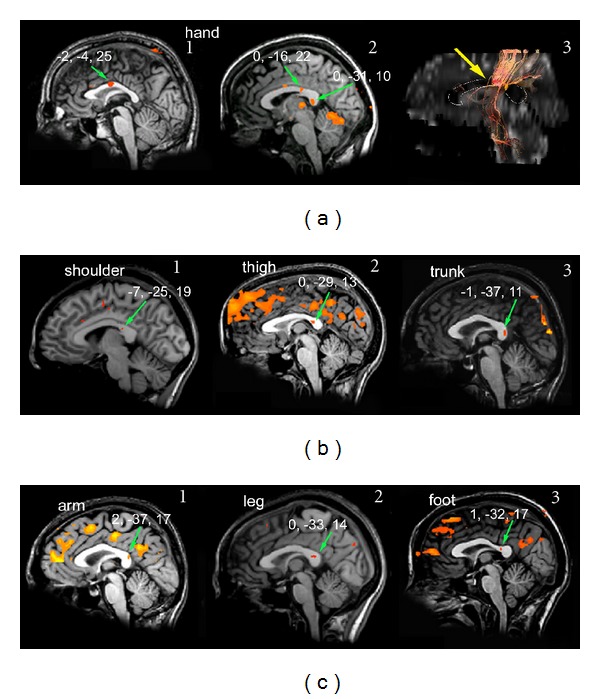
Callosal BOLD effect evoked by tactile stimulation of different body regions in control subjects. (a), CC foci evoked by tactile stimuli to the hand in subjects Ca.C. ((a)1) and M.S. ((a)2). Activation was always in the central part of the CC. Tactile hand stimulation often activated also the splenium, shown in frame 2 (subject M.S.). (b), callosal foci evoked by tactile stimulation of proximal body regions (shoulder, thigh, and trunk) in subjects I.P. ((b)1), F.F. ((b)2), and E.G. ((b)3), respectively. (c), CC foci evoked by stimulation of distal body regions (arm, leg, and foot) in subjects E.G. ((c)1), I.P. ((c)2), and E.P. ((c)3), respectively. All foci lay in the posterior callosal region. Fibers arising from the activated anterior parietal cortex cross the CC through its central portion ((a)3, yellow arrow). On DTI image, a thin white line demarcates the CC. Numbers on each brain represent the Talairach coordinates (*x*, *y*, *z*) of each focus (green arrows).

 Unilateral tactile stimuli applied to left or right body regions (Table 1) activated the primary (SI; [35, 36]) and secondary (SII; [37]) somatosensory areas in the parietal cortex in all 22 subjects. They also evoked callosal foci, more frequently in the middle CC and in the posterior callosal body (Figure 4). Stimuli applied to the hand and foot evoked a BOLD focus in the middle of the CC (Figure 4(a)), while stimulation of proximal body regions (trunk, shoulder, and thigh) activated fibers in more posterior regions (Figure 4(b)). Stimulation of the arm, foot, and leg also activated posterior fibers (Figure 4(c)) that were not, however, clearly distinguishable from those activated by the stimulation of proximal body areas. In some cases, multiple foci were elicited by stimulation of a single region (e.g., hand, 4 subjects; tongue, 1 subject; foot, 1 subject; Table 3). The *y* Talairach coordinate ranged from 8 to −9 for anterior foci and from −21 to −36 for posterior foci. Mean values are reported in Table 3. In these subjects interhemispheric fibers crossing through the CC (Figure 4(a)3) were also detected at sites harboring CC foci.

**Figure 5:**
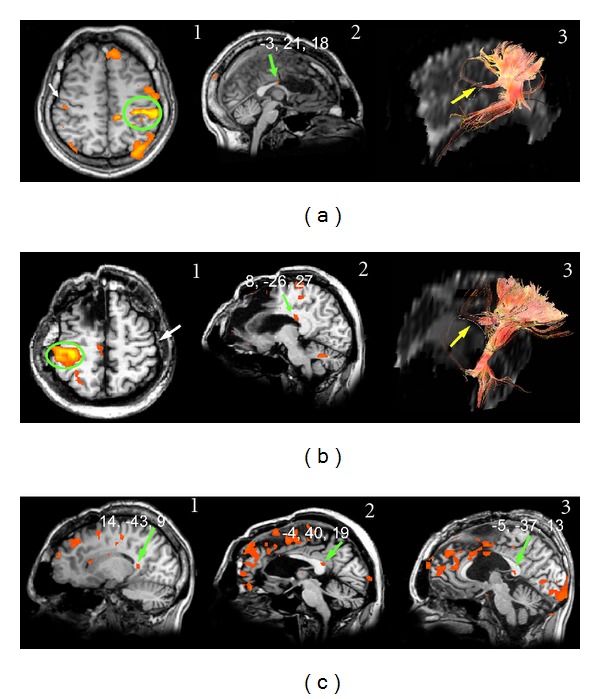
Activation evoked by tactile stimulation of the hand in patients L.M. (a) and O.T. (b). Activation of the anterior parietal cortex is seen in both patients ((a)1 and (b)1), but it is bilateral in L.M. ((a)1). Callosal activation is seen in both patients ((a)2 and (b)2, green arrows). Fibers arising from the activated cortex cross through the central CC ((a)3 and (b)3, yellow arrows). (c) Activation foci evoked in the splenium by tactile hand stimulation in patients L.M. ((c)1), O.T. ((c)2), and P.F. ((c)3). On DTI images, a thin white line demarcates the CC. Numbers on each brain represent the Talairach coordinates (*x*, *y*, *z*) of each focus (green arrows). White arrows mark the central sulcus. Left hemisphere on the right.

Unilateral tactile stimuli applied to the left or right hand (Table 2) activated both SI and SII in the contralateral parietal cortex [14] in all 6 patients, as in control subjects. In addition, the 3 patients whose PCB was spared exhibited different activation patterns that included bilateral activation of the posterior parietal (PP) cortex and SII (L.M.; Figure 5(a)1); bilateral PP cortex activation (O.T.); bilateral SII activation (P.F.; not shown). In the 3 patients with callosotomy involving the PCB activation was only contralateral. Tactile stimuli also evoked callosal foci, more frequently in the PCB, in patients in whom the central CC region was spared (L.M., O.T., and P.F.; Figures 5(a)2 and 5(b)2). The *y* coordinate ranged from −19 to −26 (see mean values in Table 2), very similar to that observed in control subjects (see above; [29]). In the patients with anterior callosotomy interhemispheric fibers crossing through the CC (Figures 5(a)3 and 5(b)3) were also detected at sites harboring CC activation foci. O.T. exhibited additional foci, one in the anterior callosal midbody (not shown) and another in the splenium, while L.M. and P.F. showed a focus in the splenium (Figure 5(c); Table 4).

*Auditory Stimulation (1 Normal Subject and 3 Callosotomy Patients)*

**Figure 6:**
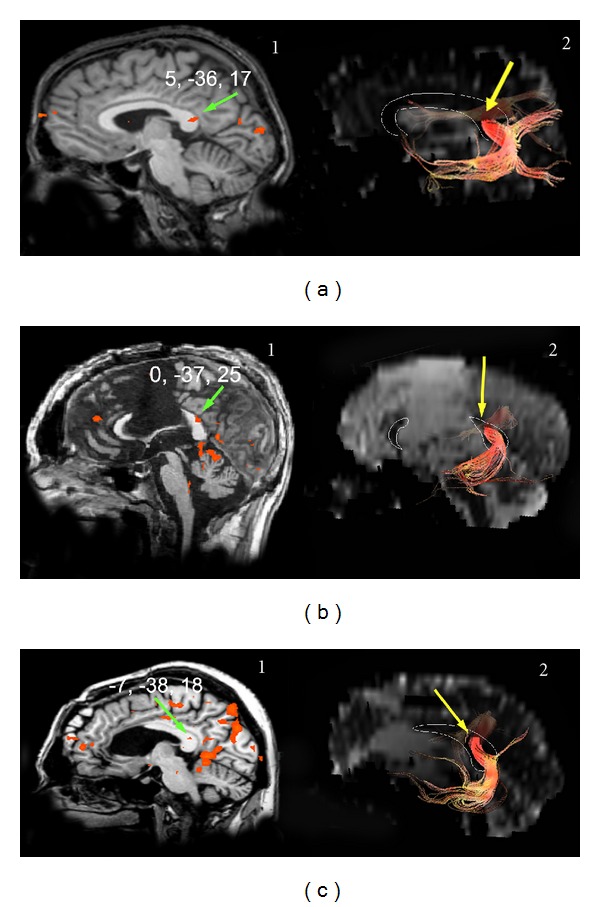
Activation evoked by auditory stimulation in control subject E.L. (a) and in patients P.F. (b) and O.T. (c). Callosal foci are visible in the control subject and in both patients ((a)1, (b)1, and (c)1, green arrows). Fibers arising from the activated cortex cross through the posterior CC, likely the isthmus ((a)2, (b)2, and (c)2, yellow arrows). On DTI images, a thin white line demarcates the CC. Numbers on each brain represent the Talairach coordinates (*x*, *y*, *z*) of each focus (green arrows).

Monaural (music) or binaural (words) stimuli induced activation in the primary auditory cortex and in the posterior CC (isthmus/splenium) in all participants (Figure 6). The values of the *y* coordinate were −36 in the control subject, and ranged from −35 to −38 in patients. Mean control values are reported in Table 3, and mean patient values in Table 4. Interhemispheric fibers connecting the activated auditory areas crossed through the isthmus in the control subject and the spared splenium in patients (Figure 6) at the same sites harboring the BOLD foci.

*Visual Stimulation (14 Normal Subjects and 5 Callosotomy Patients)*

**Figure 7:**
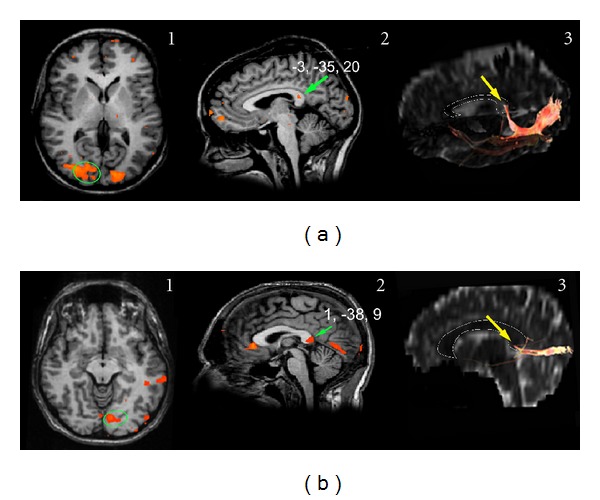
Callosal BOLD effect evoked by visual stimulation in control subjects. (a) Activation was evoked by visual stimuli presented to the CVF (subject E.N.), with a BOLD effect being consistently observed bilaterally in area VI ((a)1) and in the posteriormost part of the splenium ((a)2, green arrow). Callosal fibers arising from an ROI selected in area VI ((a)1, green circle) cross the CC through the splenium, where the activation focus is found ((a)3, yellow arrow). (b) Activation evoked by visual stimuli presented to the lateral VF (subject A.Q.). Cortical activation was present only on the contralateral side ((b)1) in a slightly more posterior splenial region ((b)2, green arrow). Callosal fibers arising from an ROI selected in area VI ((b)1, green circle) cross the CC through the splenium, where the activation focus is observed ((b)3, yellow arrow). On DTI images, a thin white line demarcates the CC. Numbers on each brain represent the Talairach coordinates (*x*, *y*, *z*) of each focus (green arrows). Left hemisphere on the right.

**Figure 8:**
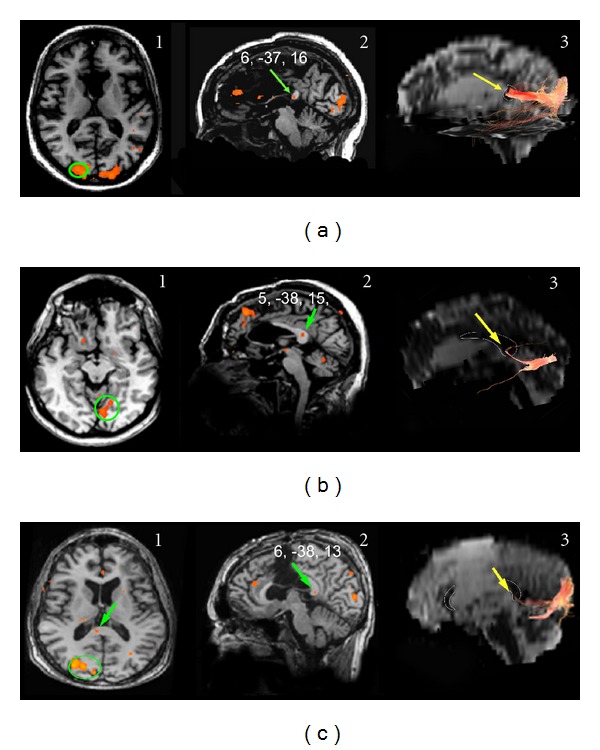
Activation evoked by visual stimulation in callosotomy patients. (a) CVF stimulation in patient D.B. Cortical activation is seen in the occipital cortex of both hemispheres ((a)1) and in the splenium ((a)2, green arrow). Fibers arising from the activated cortex ((a)1, green circle) cross the CC through the extant part of the splenium ((a)3, yellow arrow). (b) and (c) Activation evoked by peripheral visual stimulation in patients O.T. (b) and P.F. (c). Cortical activation is seen in the occipital cortex of the contralateral hemisphere ((b)1 and (c)1) and in the splenium ((b)2 and (c)1 and 2, green arrows). Fibers arising from the activated cortex ((b)1 and (c)1, green circles) cross the CC through the spared portion of the splenium ((b)3 and (c)3, yellow arrows). Note that in VI the fiber bundle arising from the representation of the peripheral retina and crossing the splenium is much thinner than the bundle arising from the foveal representation, pictured in (a). On DTI images, a thin white line demarcates the CC. Numbers on each brain represent the Talairach coordinates (*x*, *y*, *z*) of each focus (green arrows). Left hemisphere on the right.

Unilateral visual stimuli, presented to the LVF, RVF, or CVF (Table 1), elicited activation foci in the primary visual cortex and in the splenium in all 14 subjects (Figure 7(a) for CVFs, Figure 7(b) for peripheral VF). The values of the *y* coordinate ranged from −30 to −42. Mean values are reported in Table 3. Foci evoked in the splenium by CVF stimulation seemed to be slightly more anterior than those evoked by LVF stimulation, but the difference was not significant. The interhemispheric fibers linking the activated visual areas crossed through the splenium (Figures 7(a)3 and 7(b)3) at sites harboring the BOLD foci. Also in patients unilateral visual stimuli presented to the LVF, RVF, or CVF (Table 2) induced activation foci in VI in all 5 patients (Figure 8(a) for CVF stimulation and Figures 8(b) and 8(c) for peripheral VF stimulation). VI activation was usually bilateral after CVF stimulation, and contralateral after peripheral VF stimulation. In patients whose splenium was spared an activation focus was seen in this region (Figure 8, central column). The value of the *y* coordinate ranged from −32 to −38 (mean values in Table 3), consistent with control values (mean −35; [29]). The interhemispheric fibers linking the activated visual areas crossed through the extant splenium (Figure 8, right column) at sites harboring the BOLD foci.

Multiple foci were quite often elicited by peripheral stimulation in control subjects (Table 3), namely, in 4 subjects receiving tactile hand stimulation, in one receiving tactile stimulation of the tongue, in one receiving foot stimulation, in 2 subjects receiving bitter taste stimulation, and in one subject receiving the sweet stimulus. Subjects showing double foci for tactile and sweet stimulation of the tongue also displayed multiple activations after tactile stimulation of the hand. These multiple foci are shown in Table 3 and Figures 1 (taste) and 5 (touch). Two foci could also be observed in patients, particularly after taste and touch stimulation; one usually lays at the site corresponding to the sensory stimulus applied (i.e., in the anterior CC after taste stimulation, in the mid-posterior CC after touch stimulation), whereas the other was found in the splenium (Figures 2(b), 2(c), and 5(c)).

Statistical analysis with Wilcoxon's test showed that the *y* coordinates of the foci evoked by visual stimulation were significantly different (*P* ≤ 0.01) from those elicited by all the other modalities, sensory as well as motor. The foci evoked by tactile hand stimulation were different (*P* = 0.01) from those evoked by taste activation. Hand motor stimuli evoked foci at CC sites that were significantly different from those evoked by hand touch stimuli in the posterior CC, but not from those elicited in the anterior CC. The anterior and posterior gustatory foci, if considered separately, were both significantly different from those elicited by CC motor activation (*P* ≤ 0.05).

### 3.2. Tractography

Data for DTI and DTT processing were obtained from 17 control subjects and from all 6 patients. FA values in the CC regions crossed by fibers interconnecting activated areas were similar to those reported in previous studies [38–40] and were not significantly different among callosal regions. In particular, FA was 0.72 in the genu of control subjects and 0.68 in the spared genu of patients; 0.69 in the PCB of normal subjects and 0.61 in patients' PCB; 0.68 in the splenium of controls and 0.73 in the extant splenium of patients [39, 40]. The FA difference between the same callosal regions in controls and patients was never significant.

*Callosal Fibers Arising from Individual Cortical ROIs*

Examination of the fibers arising from a cortical ROI selected in area GI showed that they crossed through the anterior part of the CC (genu), that is, the region activated by taste stimuli, both in controls (Figure 1(e)) and in patients with intact genu (M.C. and P.F.; Figure 2(a)2). No fibers from area GI were seen to cross through the CC at the level of the splenium. Fibers from an ROI selected in the mediolateral frontal cortex activated by motor stimulation of the hand crossed through the CC slightly more posteriorly (Figure 3(b)). Fibers from an ROI selected in the mediolateral parietal cortex activated by tactile stimulation of the hand crossed through the CC even more posterior in controls (Figure 4(a)3) and in patients with this callosal region preserved (L.M., O.T. and P.F.; Figures 5(a)3 and 5(b)3). Analysis of an ROI selected in the primary auditory area showed fibers crossing through the isthmus/splenium, at a site activated by auditory stimulation, both in the unique control subject and in patients (Figure 6). Finally, analysis of an ROI selected in the activated region of VI showed fibers crossing through the splenium, at a site activated by visual stimuli, both in controls (Figure 7) and in patients (Figure 8).

*Callosal ROIs*

When ROIs were selected in the genu, anterior body, PCB, isthmus, and splenium harboring activation foci, the callosal fibers were seen to interconnect, respectively, the frontoparietal opercula and prerolandic, parietal, temporal, and occipital regions, which harbored foci activated by taste stimulation of the tongue, hand motor tasks, tactile stimulation of the hand and trunk, and visual stimulation. These findings regarded both control subjects and patients [41].

Even though activation foci were seen in the splenium of some patients after taste or touch stimulation, no interhemispheric fibers from gustatory or somatosensory cortical areas were seen to cross through the CC at this level; similarly, no callosal fibers coursed from the foci in the splenium to cortical regions other than occipital or temporal areas.

Finally, no correlation was observed between the FA values of the different callosal regions and the occurrence of the activation foci.

## 4. Discussion

In this paper we review our data showing the callosal activation evoked by a variety of peripheral sensory stimuli in a group of normal subjects (controls) and in 6 partial callosotomy patients. Altogether these findings show that (i) a callosal BOLD effect can be evoked by peripheral sensory stimulation and by motor tasks other than interhemispheric transfer tasks, (ii) CC activation foci occupy consistent locations that are related to the sensory or motor stimulus applied, and (iii) the topographical map of the CC thus obtained is in line with human postmortem data [8], with the investigations of patients with CC injury or surgical resection [9, 14, 15, 42–46] see ([10, 11], for a review), and with electrophysiological recording and neuroanatomical animal studies [1–3, 5–7].

### 4.1. Basis of Callosal Activation

To date, a callosal BOLD effect has been described in relation to visual and motor stimulation [20–24, 47] and, recently, in response to simple sensory stimuli [29]. Energy-dependent processes also take place in WM, since they are often axon conduction mediated (at the level of the nodes of Ranvier) by adenosine triphosphate-dependent Na^+^-K^+^ ion pumps that restore ionic gradients across the neuron membrane after action potential propagation [48, 49]. Moreover, it has been shown that the inhibition of voltage-dependent Na^+^ channels suppresses the fMRI response to forepaw somatosensory activation [48]. In addition, according to recent evidence, spiking activity is also correlated with fMRI activation [50–52]. The notion of a BOLD effect in WM is therefore becoming accepted. It has tentatively been explained with the involvement of astrocytes [53, 54] acting on vessel dilation to meet the greater metabolic demand from the heightened activity, which in turn results in increased neurotransmitter release in the extracellular environment, in raised K^+^ levels in the extracellular medium due to augmented neural activity, and/or in increased cytoplasmic Ca^++^ [53, 55]. Both astrocytes and capillaries are found in the commissure [56], and since callosal axon fibers need energy to conduct action potentials, the mechanism is likely active in CC fibers too. According to another—purely physical—hypothesis the heat generated by the increased axonal metabolic activity would be sufficient *per se* to produce callosal microvessel vasodilation (LeBihan, 2009, personal communication).

### 4.2. Topography of Callosal Activation

The foci evoked by different types of sensory stimuli and by motor stimulation occupied different locations along the commissure. Apart from some exceptions that will be addressed below, the topographical distribution of the callosal activation foci was consistent with the functional organization of the commissure as emerging from anatomical and neuropsychological studies. In our subjects foci induced by taste stimuli lay mainly in the anterior CC, that is, the genu and anterior body, which also harbored foci elicited by tactile stimulation of the tongue. The overlap is likely due to the complex nature of taste, which includes a tactile component.

**Figure 9:**
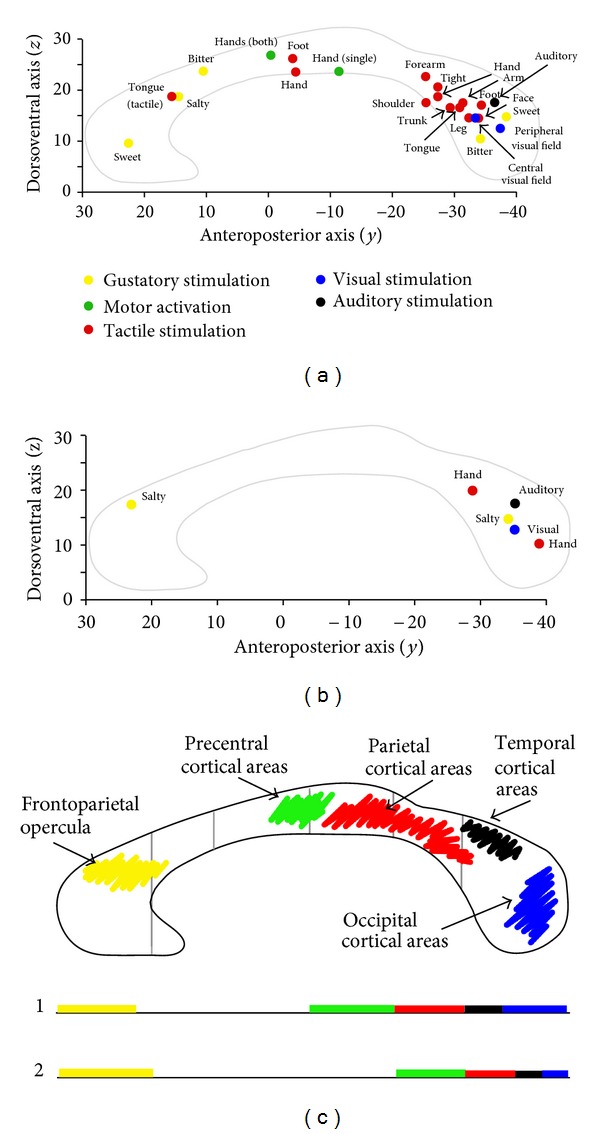
Summary schematic diagram showing the similar distribution of the callosal activation foci evoked by different stimulus types in controls (a) and patients (b). In (c) the distribution of callosal foci is compared to that of the crossing site of callosal fibers seen in our work and in the studies of Witelson [57] (gray lines), Hofer and Frahm (line 1, [58]), and Chao et al. (line 2, [59]). (a and b). Each dot represents the “mean” value of the *y* and *z* Talairach coordinates (reported on the respective Cartesian axes) of the foci evoked by different stimuli. Yellow: foci evoked by gustatory stimuli; green: foci evoked by hand motor tasks; red: foci evoked by tactile stimuli; black: foci evoked by auditory stimuli; blue: foci evoked by visual stimuli. See the text for a detailed description. (c) shows the crossing sites of interhemispheric fibers connecting the sensory and motor cortical areas activated by the relevant peripheral stimuli. Vertical gray lines mark the main CC subdivision according to Witelson [57]. Line 1 and line 2 on the bottom show the CC subdivision according to Hofer and Frahm (Figure 3 of [58]) and Chao et al. (Figure 7 of [59]): colored tracts mark the CC crossing sites of fibers from frontal opercular cortical areas (yellow), motor cortices (green), anterior and posterior parietal cortices (red), temporal cortices (black), and occipital cortices (blue).

The foci evoked by hand motor stimulation and by tactile stimuli of different body regions were seen in the anterior and posterior body and in the isthmus, respectively. In particular, proximal body representations seem to be connected by callosal fibers running through the posterior isthmus and anterior splenium (Figure 9). A dorsoventral topographical organization could not be recognized.

As expected, foci evoked by auditory and visual stimuli were found in the callosal region corresponding to the isthmus/splenium and splenium, respectively. Callosal foci activated by central or peripheral VF stimulation lay at sites whose coordinates were not significantly different.

Unlike previous investigations of the callosal BOLD effect, the sensory stimuli and motor tasks employed in our studies did not require interhemispheric transfer. CC activation was nonetheless observed, suggesting that all information reaching a cortical area is likely transferred to the opposite hemisphere and used to build a continuous representation of the external world.

As noted above, activation was sometimes found in unexpected callosal regions. This is the case of a posterior focus, which was often evoked by taste stimuli in some controls and patients in addition to the anterior foci seen in all subjects. Similarly, tactile stimulation of the hand and foot elicited a focus in the anterior part of the posterior body in the CC regions activated by the hand motor tasks; in addition, stimulation of the hand produced a focus in the splenium in some control subjects and in some patients. The first explanation that comes to mind, that which the available data cannot, however, rule out, is that the “ectopic” foci are artifacts. However, since their presence does not appear to be accidental, they are in fact probably related to the activity evoked by peripheral stimuli. Thus, the anterior focus observed after tactile hand and foot stimulation could be associated with motor cortex activation, which is often induced by tactile stimulation of these regions [35]; the foci evoked in the splenium by taste and hand tactile stimuli might result from concurrent activation of high-order association areas, like the posterior parietal cortex (PPC) in the case of tactile stimuli, and/or temporal areas for both taste and tactile stimuli; the fact the PPC and temporal areas are connected by fibers crossing though the splenium [58, 59] may explain those findings.

### 4.3. Correspondence with Tractography Data

The functional callosal topography sketched by the foci described herein, apart from the exception mentioned above, is in line with the topographical organization of CC fibers described in previous anatomical studies [25, 58–61]. The fibers connecting the prefrontal cortical areas were seen to cross through the anterior part of the CC, those connecting the premotor and motor cortical areas crossed at the level of the central callosal body (see also [62]), those connecting the parietal cortical areas crossed through the posterior callosal body, and the fibers linking the occipital and temporal areas crossed at the level of the splenium [38, 63]. In all our tests and subjects, the fibers arising from the cortical areas activated by each stimulus type crossed through the CC in a region corresponding to the one harboring the relevant callosal focus/i. The coincidence of callosal BOLD activation and crossing point of the DTT-reconstructed interhemispheric bundles (Figure 9) strongly suggests that the CC foci evoked by sensory stimulation and motor tasks may be due to the activation of the fibers connecting the activated areas to the corresponding contralateral areas and carrying specific information. In all control subjects and in patients the presence of callosal foci in the spared callosal regions broadly corresponded with bilateral activation of the cortical areas reached by specific unilateral peripheral sensory input. Although an exact correspondence cannot at present be demonstrated due to technical limitations (i.e., BOLD and DTI data cannot be coregistered and shown in the same image), we are convinced that it does exist, at least based on the consistency of the observations.

### 4.4. Comparison with Other Studies

The present outline of CC topography is based on the study of fibers arising from different sensory areas activated by relevant peripheral stimuli. With regard to taste we analyzed different submodalities (tastants); in the touch modality we studied discrete body representations, and in the visual modality we explored the activation evoked by peripheral and central visual field stimulation. With regard to auditory stimulation our data are still confined to a small number of subjects and stimuli, while motor activation was only analyzed in controls. We decided to include the data from all, even small samples, because together with those from larger samples they may help provide a more comprehensive picture of callosal functional topography.

The main difference between previous human after mortem and DTI studies and monkey neuroanatomical tracing investigations is that the topographical map outlined by our data is less precise. In brief, we have two sets of data one of which agrees with several studies from other laboratories; the other set is ostensibly in contrast with previous knowledge. Often, multiple foci (usually two) were activated by the stimulation of a single body region. This happened for tactile hand stimulation and sometimes also for taste and tactile foot stimulation. The multiple foci contrast with the single callosal sites identified in previous DTT studies [58–62]. The discrepancy might be due to the fact that the natural peripheral stimulation applied in our fMRI studies may have involved a larger number of areas than primary sensory and/or motor cortical ones, whereas DTI and monkey neuroanatomical studies address the trajectories of fibers arising in circumscribed cortical areas. In particular, tactile stimulation of the hand or foot evokes activation in the primary motor cortex [35], which could anticipate an action of the limb receiving the tactile stimulus, explaining a subsequent callosal activation (as in interhemispheric transmission aimed at motor output coordination). Splenium activation could also account for the good performance of callosotomy patients in whom only the splenium survives in tasks involving interhemispheric transfer of tactile information, a commonly reported observation [12, 64, 65]. It may be therefore hypothesized that the splenium is involved in the transfer of touch information, providing for a degree of plasticity in patients with callosal partial resection. Further evidence from neuropsychological studies of taste sensitivity in callosotomy patients [66, 67] points to a role for the splenium in transferring taste information. The splenial callosal foci elicited by unilateral taste stimulation in our studies lend support to this notion. An involvement of the splenium in the transfer of information other than visual sensory data could be due to the prominent role of the visual representation of the external environment, typical of humans, where sensory experience is usually associated with a visual component.

Previous papers describing callosal activation reported a BOLD effect in the anterior portion of the CC (see data and literature in [68]). Fibers interconnecting prefrontal and motor cortical areas course through this region and are likely involved in behavioral visuomotor interhemispheric transfer tasks, like those evoking callosal activation in those studies. Anterior CC activation has been hypothesized to be related to the involvement of fibers carrying interhemispheric information between these cortical areas. The data reviewed in the present study were obtained from simple sensory stimulation that did not involve a motor output, and from simple motor tasks. This may explain the WM activation seen in regions where sensory or motor fibers cross through the commissure.

## 5. Conclusions

In the present paper we have reported the data collected in normal subjects (controls) and in some callosotomy patients during fMRI studies on peripheral sensory stimulation. It has been shown that a BOLD effect can be evoked in the corpus callosum by peripheral sensory stimulation and by motor tasks and that CC activation foci occupy consistent locations related to the sensory or motor stimulus applied; it has thus emerged that a functional topographical map of the CC is in line with previous investigations. Further studies, however, combining fMRI and DTI, are needed to provide a better understanding of the topography of callosal activation and to confirm or exclude its correspondence with fiber crossing. It would be also interesting to clarify if callosal fibres carrying information about different submodality or different regions of the sensory periphery also follow any particular organization. As pointed out by Pandya and Seltzer “The understanding of the precise topography for commissural fibers allows one to assess the nature of functional deficits following selective commissural lesions and to predict the localization of functions within cerebral commissural systems” [7].

## Figures and Tables

**Table 1:** Summary of subjects studies and stimulation sites.

Subject					Tactile										Gustatory						
	Age	Gender	Old field score	DTI	Tongue	Face	Hand	Forearm	Arm	Shoulder	Trunk	Thigh	Leg	Foot	Salty	Sweet	Neutral	Bitter	Visual	Motor hand	Auditory
I.P.	22	F	18 (right)	yes		L	L			L	L		L	L							
E.P.	24	F	19 (right)				L				L			L							
A.I.	35	M	13 (right)				R	R		R		R		R							
F.F.	23	F	13 (right)			L	L	L			L	L		L							
Ca.C.	30	M	19 (right)				L*	L					L								
E.G.	24	F	10 (right)				L*		L		L		L								
A.M.	24	M	16 (right)						L	L		L	L								
L.A.	23	M	26 (right)				L				L					L, R	L, R				
Al.M.	28	F	10 (right)		L, R	L, R									L, R		L, R			L&R	
G.M.	33	F	46 (left)	yes		L, R									L, R			L, R		L&R	
A.V.	23	M	16 (right)												L, R		L, R			L&R	
Ch.C.	27	F	10 (right)												L, R						
E.B.	27	F	14 (right)		L, R										L	L, R	L				
M.P.	36	M	11 (right)												L, R						
M.R.	25	M	13 (right)												L, R		L, R				
M.S.	28	F	41 (left)		L, R										L, R	L, R	L, R				
Lu.A.	38	F	10 (right)		L, R										L, R	L, R	L, R				
Is.P	30	F	19 (right)				L									L, R		L, R			
Y.H.	27	F	11 (right)				L&R											L&R	L&R		
Ci.M	26	F	12 (right)				L												L&R		
C.G.	37	F	17 (right)																L		
A.Q.	28	M	10 (right)	yes															L, R		
M.F.	47	F	11 (right)																L, R		
F.S.	28	F	50 (left)	yes			R													L	
D.C.	22	M	10 (right)	yes			L, R													R	
A.S.	43	F	10 (right)	yes			R	L				L								L	
M.A.	50	M	10 (right)	yes					L			L								L, R	
G.C.	35	M	10 (right)	yes															C		
I.C.	28	F	22 (right)	yes															C		
Igo.C.	33	M	17 (right)	yes															C		
M.B.	46	M	10 (right)	yes															C		
E.N.	29	M	10 (right)	yes															C		
B.N.	54	M	10 (right)	yes															C		
M.D.P.	42	F	40 (left)	yes						L				L					C		
E.A.	36	M	13 (right)	yes				L		L									C		
L.P.	51	F	11 (right)	yes															C		
E.L.	26	M	18 (right)	yes											L, R	L, R					L&R

*Back of the hand.

DTI: 17 subjects.

Tactile stimulation: 22 subjects.

Gustatory stimulation: 13 subjects.

Visual stimulation: 14 subjects.

Motor activation: 7 subjects.

Auditory stimulation: 1 subject.

L: left side stimulation.

R: right side stimulation.

L, R: stimulation of the left and right side alternatively during the same session.

L&R: stimulation of the left and right side simultaneously.

**Table 2:** Summary of patients studied and stimulation sites.

						Stimulation			
Subject	Age	Gender	Oldfield score	Callosal resection	DTI	Taste	Tactile	Visual	Auditory
						Salty	Hand		
M.C.	51	M	10 (right)	Partial posterior	Yes	L, R	L, R	L, R—C	
D.B	59	F	11 (right)	Partial anterior	Yes		L, R	L, R—C	
R.V.	39	M	10 (right)	Partial anterior	Yes	L, R	L, R	L, R—C	
L.M.	32	F	10 (right)	Partial anterior	Yes	L, R	L, R		L&R
O.T.	51	M	10 (right)	Partial anterior	Yes		L, R	L, R	L, R
P.F.	26	M	10 (right)	Partial central	Yes	L, R	L, R	L, R	L, R

L: left side stimulation.

R: right side stimulation.

L, R: stimulation of the left and right side alternatively during the same session.

L&R: stimulation of the left and right side simultaneously.

**Table 3:** Mean Talairach coordinates of callosal activation foci evoked in control subjects by different kind of stimulation.

Stimulation	Talairach coordinates		
	*x*	*y*	*z*
Sweet anterior (*n* = 4)	3	21	12
Sweet posterior (*n* = 3)	1	−38	14
Salty (*n* = 4)	0	15	19
Bitter anterior (*n* = 2)	−4	10	25
Bitter posterior (*n* = 2)	−9	−34	10
Hand motor L&R (*n* = 3)	0	0	26
Hand motor L or R (*n* = 5)	0	−11	23
Hand anterior (*n* = 8)*	−3	−4	23
Hand central (*n* = 9)*	−1	−27	18
Face (*n* = 4)	0	−33	14
Tongue anterior (*n* = 4)	3	16	18
Tongue posterior (*n* = 2)	−5	−30	16
Forearm (*n* = 3)	0	−26	22
Arm (*n* = 3)	1	−31	17
Shoulder (*n* = 3)	−3	−25	17
Trunk (*n* = 4)	0	−29	16
Thigh (*n* = 5)	−2	−27	20
Leg (*n* = 4)	−1	−32	14
Foot anterior (*n* = 2)**	1	−3	25
Foot posterior (*n* = 4)**	−2	−34	16
Peripheral visual field (*n* = 5)	2	−37	12
Central visual field (*n* = 9)	1	−33	14
Auditory binaural (*n* = 1)	−5	−36	17

*5 subjects show activation both in the anterior and central focus.

**1 subject shows activation both in the anterior and central focus.

**Table 4:** Mean Talairach coordinates of callosal activation foci evoked in patients by different kind of stimulation.

Stimulation	Talairach coordinates		
	*x*	*y*	*z*
Salty anterior (*n* = 2)	−4	22	19
Salty posterior (*n* = 2)	−2	−35	17
Hand central (*n* = 3)*	−1	−30	22
Hand posterior (*n* = 3)*	2	−40	14
Visual (*n* = 5)	4.5	−36	16
Auditory (*n* = 3)	−1	−36	20

*all 3 patients show activation both in the central and posterior CC.
